# Ripe on time: How posttranslational modifications of a transcription factor impact tomato fruit ripening

**DOI:** 10.1093/plphys/kiae308

**Published:** 2024-05-28

**Authors:** Anna Moseler

**Affiliations:** Assistant Features Editor, Plant Physiology, American Society of Plant Biologists; INRES-Chemical Signalling, University of Bonn, 53113 Bonn, Germany

Food loss during postharvest storage is not just an economic problem but also forms a major threat to global food security. Fruits and vegetables especially are susceptible to spoilage and quality loss. One of the most important crops worldwide is tomato (*Solanum lycopersicum*), with approximately 180.8 million tons of tomato fruits being produced each year (León-García et al. 2017; Meng et al. 2022). Tomato is also used as a model plant to investigate senescence and fruit ripening because of its short life cycle and relatively small (900 Mb) genome (Tomato Genome Consortium 2012). Furthermore, an easy readout for tomato fruit ripening is the production of carotenoids, the organic pigments that give the ripe tomato its characteristic red color (Liu et al. 2015; Llorente et al. 2016). Fruit ripening is a complex process modulated by several signaling molecules like ethylene, abscisic acid, and hydrogen sulfide (H_2_S) (Alexander and Grierson 2002; Kai et al. 2019). Candidate species for the transduction of signals triggered by H_2_S are post-translationally modified proteins, called persulfides, where a cysteine thiol (-SH) is covalently bound to sulfur (-SSH) (Corpas et al. 2021).

In this issue of *Plant Physiology*, Zhang, Hu, and colleagues (Zhang et al. 2024) identified the impact of H_2_S signaling and concomitant persulfidation of the transcription factor WRKY6 on tomato fruit ripening (Fig.). First, the authors showed that increasing H_2_S levels either exogenously by fumigation or endogenously by overexpression of the cytosolic H_2_S-generating enzyme L-cysteine desulfyhdrase (LCD1) significantly delayed tomato fruit ripening. Proteomic analysis of persulfidated proteins showed a higher extent of persulfidation of the transcription factor WRKY6 in the *LCD1* overexpression lines compared with wild-type plants. This persulfidation was further verified in vitro, and cysteine 396 of the WRKY6 protein was identified as the persulfidation site. To further confirm the role of WRKY6 in senescence regulation and tomato fruit ripening, Zhang and colleagues generated *WRKY6* overexpression lines and *wrky6* null mutants. Phenotypic comparison with wild-type plants revealed that leaf senescence and fruit ripening were accelerated in the overexpression lines, while it was delayed in the null mutants. These data indicate that WRKY6 is involved in the regulation of fruit ripening and that its activity can be controlled by persulfidation.

**Figure.**
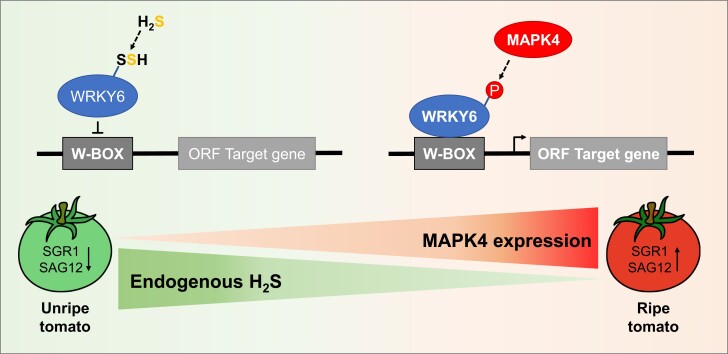
Schematic representation of the impact of posttranslational modifications on the transcription factor WRKY6 during tomato fruit ripening. In unripe tomatoes, endogenous H_2_S levels are high, resulting in the persulfidation of WRKY6. The persulfidation attenuates the binding to the W-box (WRKY-binding site) present in the promoter region of target genes, resulting in a low expression level of *SGR1* and *SAG12*. During ripening, H_2_S level decreases whereas expression of MAPK4 increases. MAPK4 phosphorylates WRKY6, leading to an enhanced binding to the W-box and increased expression of *SGR1* and *SAG12* (modified after Zhang et al. (2024)).

To further investigate the underlying molecular mechanism, the authors performed transcriptomic analysis in the leaves of *WRKY6*-overexpressing plants and found 675 upregulated and 617 downregulated genes compared with the wild type. In the overexpression lines, the chlorophyll degradation-related gene *SGR1* (*Stay-Green 1*) and the senescence-related gene *SAG12* (*Senescence-Associated Gene 12*) were significantly upregulated. Both genes contain a WRKY-binding site in their promoter, indicating that they might be direct targets of WRKY6. Furthermore, KEGG analyses revealed that several upregulated genes including *MAPK1-5* are involved in the mitogen-activated protein kinases (MAPK) pathway. The MAPK pathway plays a key role in controlling gene expression in a number of ways, including the phosphorylation and regulation of transcription factors. Hence, Zhang and colleagues identified putative phosphorylation sites of WRKY6 in silico. In vitro phosphorylation assays revealed that serine 33 of WRKY6 can be phosphorylated by MAPK4. Protein-protein interaction of WRKY6 and MAPK4 was confirmed by a pull-down assay as well as by yeast 2-hybrid and luciferase (LUC) complementation assays.

In the next part of the study, the authors explored the relation and effects of WRKY6 persulfidation and phosphorylation on its transcriptional activity. First, they showed that endogenous H_2_S content decreased, while expression of *MAPK4* increased during fruit ripening (Fig.). Next, they analyzed the transcriptional activity of WRKY6 using a dual LUC system in *Nicotiana benthamiana*. By expressing LUC under the control of the *SGR1* or *SAG12* promoter, they showed that in the presence of WRKY6, LUC activity was increased and was further enhanced in the presence of both WRKY6 and MAPK4. This enhancement was not observed when expressing a WRKY6 S33A variant lacking the phosphorylation site. On the other hand, fumigation with NaHS, a sulfide donor, resulted in a significant decrease in LUC activity, while expression of *MAPK4* in a WRKY6 C396A variant lacking the persulfidation site showed an enhanced LUC activity. These data indicate that phosphorylation of WRKY6 by MAPK4 is essential for its transcriptional activity, while persulfidation attenuates the expression of WRKY6 target genes. These observations were further confirmed by electrophoretic mobility shift assays, where Zhang and colleagues showed that persulfidation of WRKY6 diminished its ability to bind to the *SGR1* or *SAG12* promoter, whereas phosphorylation enhanced WRKY6 binding to the promoters of its target genes. Expression of WRKY6 or WRKY6 C396A in the *wrky6* background resulted in enhanced expression of *SGR1* and *SAG12* accompanied with early fruit ripening. In contrast, fruit ripening was delayed when the WRKY6 S33A variant was expressed or MAPK4 was downregulated.

In conclusion, this study established the significance of posttranslational modifications on the activity of transcription factors and the concomitant expression of target genes. Furthermore, it underpins the importance of the signaling molecule H_2_S and the subsequent persulfidation of proteins. Further studies are needed, however, to analyze how WRKY6 is specifically persulfidated and to unravel the influence of other molecules participating in fruit ripening like ethylene or abscisic acid. Nevertheless, the knowledge obtained in this study can benefit the development of strategies to boost crop conservation during postharvest storage and maintain fruit nutritional value.
